# Shelf Life of Fresh Sliced Sea Bream Pack in PET Nanocomposite Trays

**DOI:** 10.3390/polym13121974

**Published:** 2021-06-15

**Authors:** Teresa Fernández-Menéndez, David García-López, Antonio Argüelles, Ana Fernández, Jaime Viña

**Affiliations:** 1 Department of Materials Science and Metallurgical Engineering, University of Oviedo, 33203 Gijón, Spain; tere_asturias@yahoo.es; 2 SMRC Automotive Interiors Spain S.L.U., 47800 Medina de Rioseco, Spain; dgarci12@smrc-automotive.com; 3 Department of Construction and Manufacturing Engineering, University of Oviedo, 33203 Gijón, Spain; antonio@uniovi.es; 4 Klöckner Pentaplast, 33128 Vegafriosa, Spain; ana.fernandez@kpfilms.com

**Keywords:** PET, sepiolite, PA, nanocomposite, food packaging, MAP

## Abstract

Spoilage of fish due to microbiological activity is one of the biggest problems found by producers to take fresh fish products to customers. It is necessary packaging improvements to be able to increase fish shelf life and, thus, be able to travel further and to keep product freshness longer at customer’s houses. In the present work, a new material is developed for fish packaging in modified atmosphere (MAP). This material is poly(ethylene terephathalate) (PET) extruded with a polyamide (PA) nanocomposite containing nanosepiolite. Here, it is shown the production procedure from laboratory to industrial scale. Permeability to oxygen and impact mechanical properties results are shown for different samples, both at laboratory and industrial processes. At the end, a material composition is chosen to produce the finale tray which will contain the sliced sea bream. Microbiological analysis is done over the packed fish, resulting is a lower microbiological count compared to a PET control sample. This means that shelf life of pack sea bream could increase from 2–4 to 7–9 days, which is very important for both producers and customers. On the other hand, trays obtained comply with European regulations in food contact materials (FCM) and, overall, they are suitable for food packaging materials.

## 1. Introduction

Food waste is an increasingly important problem nowadays. It not only affects the economy, but also the sustainability of the food system. In the UE, around 88 million tonnes of food waste are generated annually with associated costs estimated at 143 billion euros [1]. Although all actors in the food chain have a role in preventing waste, food packaging is one of the most important ones. 

Food packaging is an indispensable aid in preserving food products by prolonging shelf life and ensuring food safety, besides contributing to reduce food loss and waste. Food packaging containers must comply with several requirements such as: protect, preserve, inform, and help to sell the product packed (marketing). Besides, these containers must follow EU regulations for food contact products [2], including good manufacturing practices [3] and European Food Safety Authority (EFSA) requirements. This industry is in continuous development, looking to reduce costs, reduce environmental impact, improve mechanical properties and increase shelf life of the products. The most widely used packaging system for fresh products has been MAP, which is generally made with a polymer container sealed with a polymeric film. MAP principle consists on the alteration of gases composition inside the packaging. The gases introduced are O_2_, CO_2,_ and N_2_, depending on the food product inside. The MAP works by inhibiting bacterial growth and oxidative reactions in the food, however the extend of preservation depends on species, initial microbial population, fat content, gas mixture, and the ratio of gas volume to product volume, and storage temperature [4].

Polymers, coming from petroleum or from natural sources, have been widely used as food packaging materials for many decades [5,6], being PET one of the most used for all kinds of foodstuff [7]. However, PET has its limitations in permeability, needing the use of multilayer materials, which adds complexity to the production process, as well as to its recyclability. Since permeability of the packaging is one of the most important properties in MAP applications, it has been key to this industry, to improve barrier properties of polymeric materials. For this, nanotechnology has become of great importance in food packaging research. Many PET nanomaterials have been developed [8,9,10,11,12] but, very few of them have tried or succeeded industrially producing these nanocomposites [13]. Between clay nanomaterials, the one most studied with PET is montmorillonite [8,9,10,11], a magnesium and aluminum silicate mineral. Its structure is expandable, able of separating the distance between layers, intercalating polymer branches between them until complete exfoliation. However, in this study, sepiolite clay is used as nanomaterial. Sepiolite is a magnesium phyllosilicate (Mg_8_Si_12_O_30_(OH)_4_(OH_2_)_4_·nH_2_O; n ≤ 8) with microfibrous morphology in one direction and two dimensions at the nanometric range [14]. The clay vary between 0.2 and 3 µm in length, 10–30 nm in width, and 5–10 nm in thickness, which gives a sepiolite a high aspect ratio of about 27. In addition, its structural formula leaves a significant number of silanol (Si-OH) groups present at the surface of the sepiolite [15], that together to a great surface area (300 m^2^g^−1^) make this clay perfect for surface modification, which is needed to enhance polymer-clay compatibility. In order to improve PET/sepiolite nanocomposite properties such as permeability and with the aim of minimizing PET degradation due to water within the sepiolite structure, in the present work, nanocomposites of PA and sepiolite are produced and then, introduced in a PET extruder as masterbatches. 

The aim of this work is to produce PET and PA/sepiolite nanocomposites to be used to fabricate trays capable of containing fresh fish and increasing its shelf life. Fish is one of the most perishable foods in the markets [16]. Immediately after fish death microbially induced activities start to develop. Thus, the purpose of this work is to produce, industrially, trays accomplishing all food packaging requirements and, at the same time, extending shelf life of the products packed. The benefits associated, for both producers and customers, to a longer shelf life are quite obvious, however, it is also very important to improve sustainability of food chain.

Microbial and migration analysis were done at the end of this work to prove the viability of using this material for food packaging. 

## 2. Materials and Methods

PET pellets from Novapet S.A. (Zaragoza, Spain) with an intrinsic viscosity (IV) of 0.81 dL/g, were kindly supplied by LINPAC Packaging (Pravia, Spain). PET-EVOH-PE laminated sheet is a PET sheet laminated with EVA/PA/EVOH/PA/PE flexible film. The sealing film to close trays made with this material was a PE film, both were also supplied by LINPAC Packaging. The sealing top used with the nanocomposite trays was a biaxially-oriented polyethylene terephthalate film (BOPET) coated with aluminum oxide (AlOx), Mylar^®^ 850 from DuPont Teijin Films UK Limited (Middlesbrough, UK). The PA used for the nanocomposites is MXD6_s-6007 (MXD6 from now on in this paper) from Mitsubishi Gas Chemical Company Inc. (Dusseldorf, Germany). It is an aromatic PA, amorph and has a very good affinity with PET matrix. Besides, it is transparent, and it is specially used to improve permeability properties [17], which is a plus when talking about food packaging materials [10,11]. Two types of sepiolite were supplied by Tolsa S.A. (Madrid, Spain), one modified with 2% of 3-metracyloxypropil trimetoxysilane (MEMO, CAS 2530-85-2) and the other one with 2% of 3-aminopropyltriethoxysilane (AMEO, CAS 919-30-2). Both organo-modifiers are suitable for food packaging with restrictions regarding the amount of absorbed substance by kg of product packed, as stayed in Regulation EC 975/2009 [18] for MEMO (0.05 mg by Kg of packed product) and in Directive 2007/19/EC [19] for AMEO (between 0.05 mg and 3 mg by Kg of product in the package).

### 2.1. Methods

The aim of this work is to produce sepiolite nanocomposite trays, using PA_MXD6 as clay carrier and PET as the main matrix. For this, the processing is divided in two parts: the first one being done at laboratory scale, and the second one developed in industrial machines. 

#### 2.1.1. Laboratory Process

Using laboratory equipment, several materials were produced in order to find the best one to take to industrial production.

First, the PA and nanosepiolite (nS) masters were produced through compounding using a twin-screw extruder (MICRO 27 GL-36D from LEISTRITZ A.G., Nürnberg, Germany); which has a maximum flow of 30 Kg/h and L/D of 36. The materials were produced at two different extruder speeds (80 rpm and 160 rpm) in order to analyze its effect in sepiolite dispersion into the matrix. Two concentrations of sepiolite (8% and 18%) per nanoclay type, that is sepiolite modified with MEMO and AMEO, were produced. The 18% master was used to produce the nanocomposite with 2% theorical nanosepiolite, whilst the other one was used for the 1% nanoclay.

**Figure 1:**
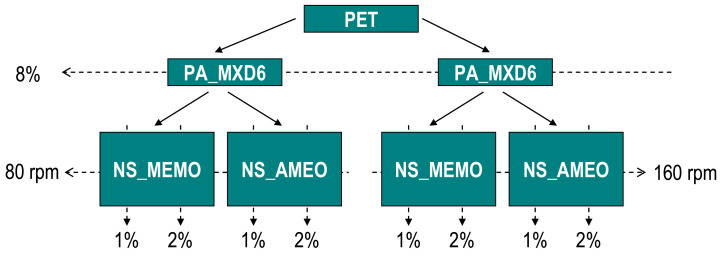
Samples composition.

The next step is to produce sepiolite nanocomposites, introducing the masters done previously into an extruder, using PET as matrix. The materials were dried prior to introducing them into the extruder. The PA_nS master drying conditions were 24 h at 80 °C, and 8 h at 120 °C for the PET. The reason for using two different drying conditions is due to the nucleating effect of sepiolite in the PA and PET matrixes, which alters their crystallization behavior [15,20]. The final composition of the materials is 91% PET, 8% PA, and 1% nS, or 90% PET, 8% PA, and 2% nS. In the figure below (Figure 1), it shows scheme with the materials produced. The percentages of sepiolite are theoretical because the extruder used did not have a special powder dosing system, so it will be seen in the results the exact amount of sepiolite in each sample. Besides these samples, a control one was produced without nanosepiolite, just using PET with 8% of PA_MXD6 (PET/8PA_MXD6).

#### 2.1.2. Industrial Processes

Once the materials obtained at laboratory scale were analyzed, the next step would be to scale up to industrial production. In the next pages, it is described the process of producing nanocomposites of PET with PA/nS industrially.

##### Extrusion and Thermoforming

The aim of this part of the process is to produce nanocomposite sheets using a corotating twin screw extruder. For this, it is necessary to obtain first of all a masterbatch of PA_MXD6 with sepiolite in order to incorporate it into the PET matrix. The process is the same as described above, in the laboratory. However, in this case the master has been produced in an industrial PA compounding extruder, in Repol S.A. facilities (Almazora - Castellón, Spain). Conditions in the production plant were optimized to minimize PET matrix degradation, reducing humidity and decreasing extrusion shear on the nanocomposites. In this step a PA master containing 20% nanosepiolite was produced (PA_MXD6/20%nS).

This master is taken to an industrial twin screw PET extruder (Luigi Bandera SpA, Busto Arsizio, Varese, Italy) using a flow between 600–1150 Kg/h, between 100 and 130 rpm and 272 °C melt temperature. The materials were previously dried using the same conditions as above (8 h at 120 °C, and 24 h at 80 °C). The sheets produced were 680 mm wide and 600 µm thick.

**Figure 2:**
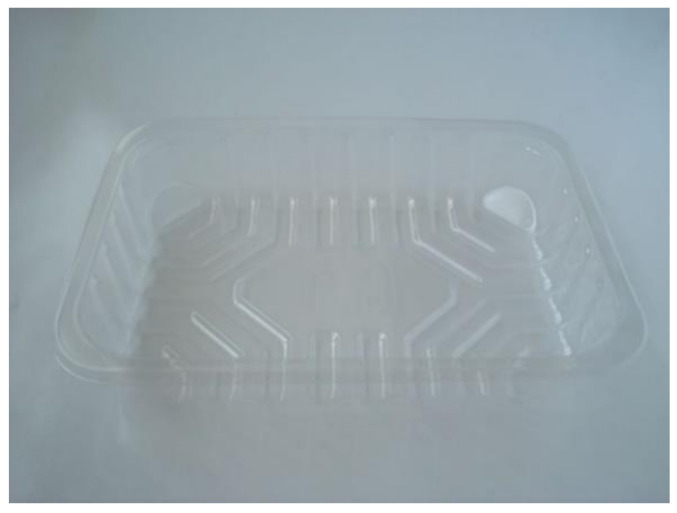
Picture of the B1825-45 tray used.

Once the sheet is produced, it is taken to the thermoforming process were the final nanocomposite trays are formed. The thermoforming machine is a KIEFEL GmbH (Sudetenstraße, Freilassing, Germany). The tray chosen was a B1825-45, which is 18 cm wide, 25 cm long and 45 mm deep (Figure 2). This is one of the most used trays for MAP of food products.

##### Thermogravimetric Analyses

Thermogravimetric Analysis (TGA) was used to determine nanosepiolite percentage within the nanocomposite sheets. The analyses were performed in a Mettler Toledo 851e equipment (Madrid, Spain), using a procedure in two steps:

1st step: from 50 °C to 600 °C at 20 °C/min under nitrogen atmosphere;

2nd step: from 600 °C to 900 °C at 20 °C/min under air atmosphere.

#### 2.1.3. Microscopy

Optical microscopy (OM) pictures have been done with an OLYMPUS BX60M microscope (Barcelona, Spain). Samples were embedded in resin and polished prior to OM observation.

Scanning electron microscopy (SEM) was performed on a fractured surface after a treatment in liquid nitrogen using a Hitachi 3400 N microscope (Krefeld, Germany).

#### 2.1.4. Permeability

The permeability analyses were done on sheet samples; specimens were taken from the extruded sheets before going to thermoforming into trays.

Oxygen transmission rate was measured in an OXTRAN with a volumetric sensor (MOCON, Oxtran SS 2/20), Barcelona, Spain. Previously to the analysis the samples were upgraded, 48 h under an atmosphere with 0% relative humidity (RH). Oxygen transmission rate was measured at 23 °C and 0% RH following Standard ASTM D3985 [21] and the effective area exposed to permeation was 50 cm^2^.

#### 2.1.5. Puncture Test

Plastic products are more prone to fail when submitted to an impact, rather than to a slow-motion load. In many applications, packaging materials are exposed to penetrating damages, which lead to barrier properties decrease and package integrity. Thus, puncture resistance is an important property in flexible packaging materials.

**Figure 3:**
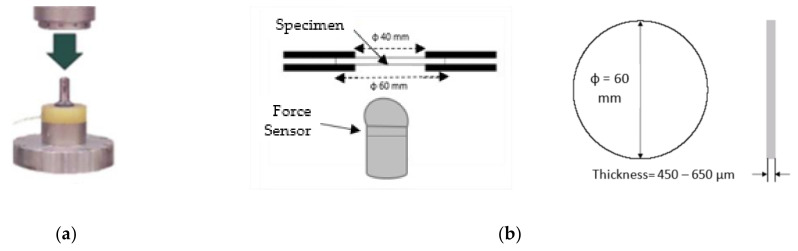
(**a**) Puncture impact scheme; (**b**) Impactor and specimen.

These impact tests were done in an MTS-831 instrumented equipment (Eden Prairie, MN, USA), following ISO 6603-2:2000 [22] methodology. The speed used was 4.4 m/s and tests were done at room temperature (23 °C). In Figure 3, it can be seen the scheme of the impact instrument and the way the specimen is hold for the impactor to hit it.

The samples were taken from the extruded sheet width, in the extrusion direction. Each specimen, with an effective diameter of 40 mm (Figure 3b), is held with two anchor rings; then the impactor (ϕ_impactor_ = 20 mm) hits on the specimen center from below (Figure 3b). The curve strength versus strain is registered for each sample, together with absorbed energy (E). However, it is very important to describe the failure mode in order to know if the material is going to break in a fragile way, a ductile, or in any of the intermediate modes in between (Table 1). In a ductile break (D), the specimen breaks slowly deforming the material with the absorbed E, while the additional, non-absorbed E, is used to extend the crease (Dc). On the contrary, in a fragile break (F) the crease is spread quickly, suddenly and totally, causing the break of the sample.

#### 2.1.6. Microbiological Tests

To do microbiological analysis three samples of each of the sea bream filets were taken. Out of these samples, the average counts are taken as samples S1 and S2 when using a PET/PA_MXD6/nS tray. At the same time, the same number of replicas have been chosen for the control samples. This time the material of the tray being PET/EVOH/PE.

For aerobic microorganism count, 25 g of superficial fish meat are taken aseptically. Samples are mixed with 225 mL of buffered peptone water and is then homogenized (dilution 1:10) in a Stomacher^®^, Seward Ltd (Worthing, Sussex, UK). After that, serial dilutions of fish homogenates were plated on the surface of the appropriate Petri dishes. The incubation time and temperature for aerobic plate count (APC) were 48 h at 30 °C [23]. For anaerobic bacteria, 10 g of fish was taken, then diluted in a 90 mL solution (0.1% peptone) and homogenized for 2 min before doing the serial dilution of samples needed. Total anaerobic bacteria were analyzed after incubations for 48 h at 48 °C [23]. Bacterial count results are expressed in log10 of colony-forming units per gram of fish meat (log cfu/g).

Microbiological analyses were done on the 12 samples the following days post packaging: 2, 4, 7 y 9. The specimens were kept at 5 °C during all that period.

## 3. Results

### 3.1. Laboratory Samples

#### 3.1.1. Thermogravimetric Analysis

Thermogravimetric analysis (TGA) results of the PET/PA/nS nanocomposites show the percentage of sepiolite in the samples (Table 2).

#### 3.1.2. Microscopic Characterization

**Figure 4:**
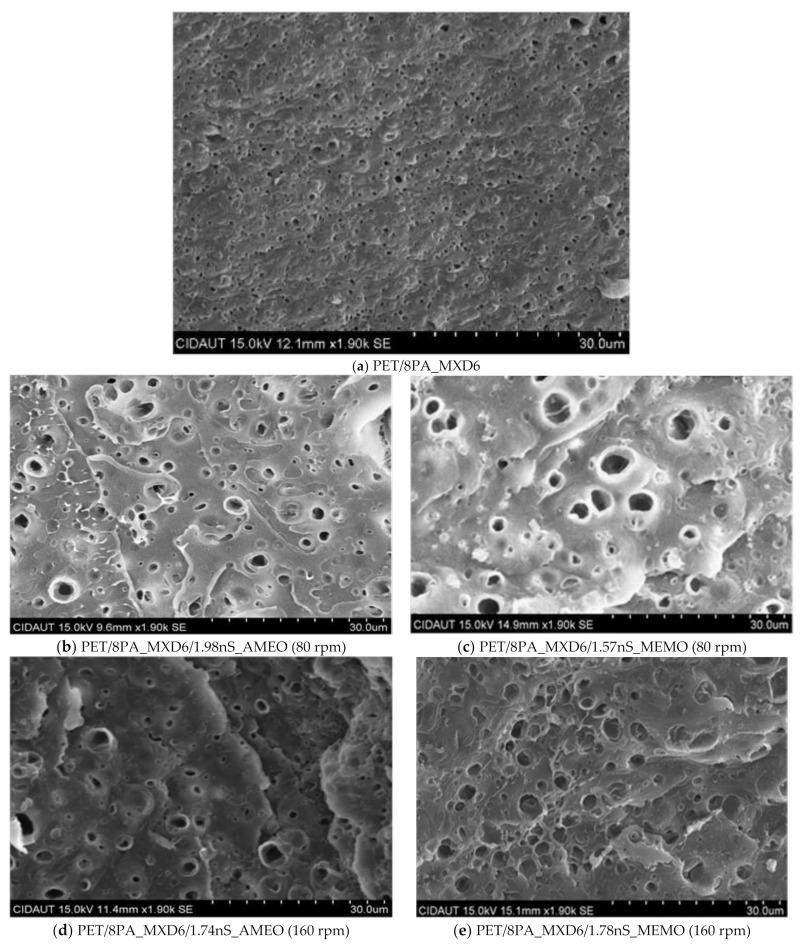
SEM pictures of PET/PA and PET/PA/nS samples with different nanosepiolite concentrations.

In Figure 4, it is shown a comparison between the different morphology of samples containing sepiolite modified with AMEO and MEMO, at the same processing speed; also, two samples AMEO modified at two different speeds but with similar nanoclay concentration (Figure 4b,d), and two samples with both sepiolite modifiers, at the same processing speed and nanoclay concentration.

SEM results show good PA_MXD6 dispersion into PET matrix, which could be attributed to the hydrogen bonding interactions between them [24]. This dispersion is slightly better PA_MXD6 particle size and distribution for samples AMEO modified than those modified MEMO. At the same time, that particle size is independent of the processing speed when using AMEO silane (Figure 4b,d), but it seems to be worse at 80 rpm when using MEMO. On the other hand, particle sizes are quite heterogeneous compared to a PET/8PA_MXD6 sample (control) (Figure 4a). Thus, nanosepiolite causes the appearance of different particle sizes populations.

**Figure 5:**
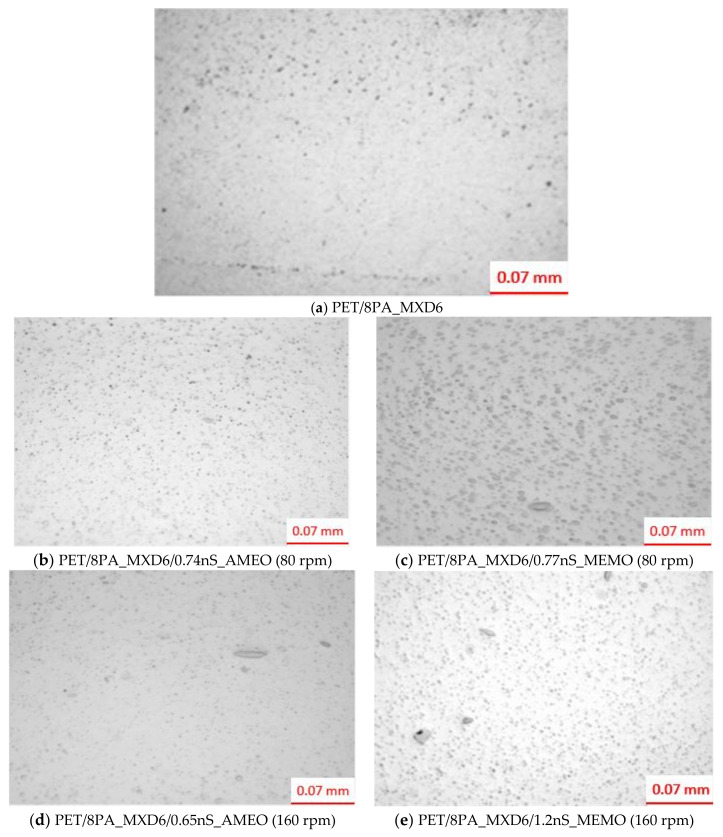
MO photomicrographs of nanocomposites PET/PA_MXD6/nS_AMEO and MEMO.

With the aim of knowing the existence of micrometric sepiolite aggregates, samples were treated to look under an optical microscope. Results show very similar particle structure in those samples whose sepiolite was either modified with AMEO or MEMO. Both specimens show micrometric aggregates heterogeneously dispersed in the samples, most of them being smaller than 30 µm. All the samples show a droplet, slightly ellipsoidal structure, in which the sepiolite is dispersed and bonded to the PA through hydrogen bonds between silanol groups of the clay and the amide groups of the PA [25]. In Figure 5, it is shown photomicrographs of nanocomposite samples produced with different screw speeds, and different silane modifier (MEMO and AMEO). It is possible to distinguish PA_MXD6 particles (rounded and smaller dots), as well as nanosepiolite aggregates (bigger and elliptical).

#### 3.1.3. Mechanical Properties

**Figure 6:**
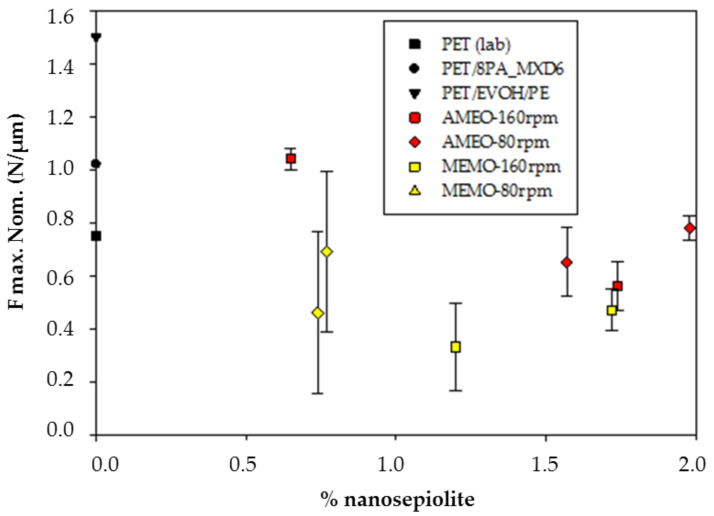
PET/PA_MXD6/nS nanocomposites Normalized Maximum Force as a function of nanosepiolite content.

When doing the puncture test, maximum force at break and perforation energy are obtained, as well as the failure type (see Table 1). When using 160 rpm to produce the masters, it has been observed a decrease in impact strength of the nanocomposites with the increase in sepiolite content. However, when producing the master at 80 rpm, F max results at this speed show high dispersion, due to fragility of the samples (Figure 6). Thus, the nanocomposites obtained have a more brittle behavior, which is in accordance with the literature [13,25,26,27,28,29]. The reasons for this fragility increased compared to the neat polymer is mainly the degradation of the PET matrix. PET can suffer degradation when processing it due to humidity content or due to the high processing temperatures. This degradation is accelerated by the presence of the organic compounds used to modify the sepiolite [29], and the longer the nanoclay stays in the extruder the worse for the nanocomposite matrix. Nevertheless, it can also be due higher crystallinity, presence of clay aggregates, and coalescence of micro voids formed around the clay particles when submitted to a force [29].

#### 3.1.4. Permeability Properties

**Figure 7:**
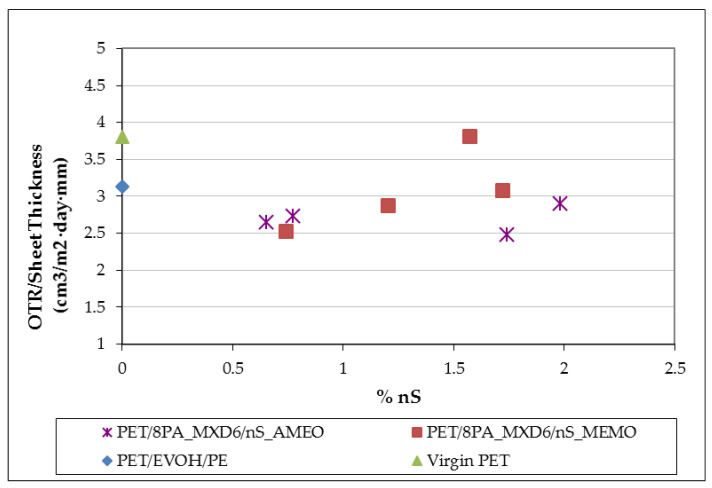
Oxygen permeability of nanocomposites.

All the samples produced with AMEO modified sepiolite have shown an improvement in oxygen transmission rate (OTR) against the reference samples, that is PET and PET/EVOH/PE sheets. This is due to the tortuous path formed by the nanoclay particles, that makes the travel through the polymer matrix, more difficult to the permeant. This improvement goes from 21% to 33% against Virgin PET, and from 9% to 22% against PET/EVOH/PE laminated sheet. However, samples modified with MEMO show poorer results in nearly all the samples. This could be due to matrix degradation [29], which causes higher permeability values. Degradation of the polymer means its molecular chains are broken, generating more free volume for the permeants. In the following figure (Figure 7), it is not shown permeability as a function of extrusion speed because it has been seen that the results are independent from this factor, and it only added confusion to the graph. When using MEMO, the tendency shows an increase in permeability when increasing nanosepiolite content, which could be due to aggregation of the clay containing particles, together with the degradation of the PET matrix. However, those nanocomposites AMEO modified keep permeability values quite constant from 0.5% nS to 2% nS. This could be due to the stability of the samples through all the production process when using this modifier. In this work, samples with sepiolite content over 2% were not produced, due to previous experiences, where it has been observed that processability of nanocomposites is much more difficult when increasing the nanoclay content [12,29].

### 3.2. Industrial Samples

The industrial proccess is highly influenced by the addtion of the PA_nS masters into the extruder. The first masterbatch used was the one modified with MEMO. When the concentrate start to enter the extruder, problems in sheet adjustment start to apear. At 30 rpm, the melt temperature was 294 °C, and the discharge pressure 110 bar. This pressure, which is an indicator of the melt viscosity, starts to decrease until 50 bar. Extrusion parameters are changed to make the temperature decrease, since that high temperature, together with the incorporation of sepiolite, contributes to the PET matrix thermal and hidrolitical degradation. After the temperature was stablished at 276 °C, it was possible to obtain a discharged pressure of 70 bar with certain variations (from 60 to 70 bar). At this moment, with an extrusion speed of 830 Kg/h, the 450 µm sheet was obtained.

After a cleaning time to stabilize the PET extruder, an AMEO masterbatch was incorporated. A decreased in discharge pressure was also observed, 80 bar, however this was lower than that produced with MEMO modified sepiolite. Temperature in this case was 275 °C. The behavior of this masterbatch in the extruder is much better, the thickness was more easily adjusted and a more stable sheet was obtained at 875 Kg/h. Being able to keep a high and constant disharge pressure and a stable sheet is very important for its final quality.

For this reason, together with the better permeability and mechanical results of the AMEO modified samples at laboratory scale, in this industrial section results of the AMEO sheets will be discussed.

#### 3.2.1. Thermogravimetic Analysis

Having into account results obtained at laboratory scale, as well as in previous works done by this group [13], the samples chosen for the industrial scale are those containing nanosepiolite modified with AMEO (Table 3 and Table 4).

#### 3.2.2. Optical Microscopy

**Figure 8:**
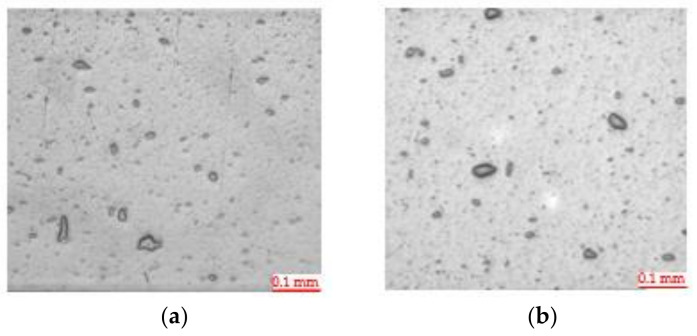
Optical microscopy of sheet samples (**a**) PET/PA_MXD6/nS_AMEO 1.1% nS, and (**b**) PET/PA_MXD6/nS_AMEO 2.1% nS.

Optical microscopy characterization has shown micrometric sepiolite aggregates with heterogeneous shapes and sizes in all the samples (Figure 8). The maximum size being close to 30 µm. Moreover, as it was seen at laboratory scale, it is possible to distinguish PA_MXD6 particles as little dots within the PET matrix, and PA_MXD6/nS as bigger an irregular shape. Sepiolite dispersion seems to be worse in industrial extruder, since the materials produced show higher number of aggregates.

#### 3.2.3. Permeability Properties

Results in Table 5 show the oxygen transmission rate (OTR) through films with different thickness. In order to facilitate comparisons among specimens OTR values were normalized to a sheet of 450 µm. It can be seen an improvement in permeability of those samples containing nanosepiolite fibers against the reference sheets (PET and PET/EVOH/PE) and, also, versus the sample of PET with PA (PET/8PA_MXD6). The best permeability is observed in sample with 2.1% of nanosepiolite, which is 43% better that PET and 34% that PET/EVOH/PET sheet. OTR of sheet containing 2.1% nS is 11% lower than that of specimen with 1.1% nS due to a more tortuous path formation.

#### 3.2.4. Mechanical Properties of Nanocomposite Sheets

Mechanical properties of food trays are very important for food producers, sellers, and buyers. Since trays need to overcome its manipulation from factory to houses, including customer’s manipulation, trays must be tough enough. Thus, it is very important to analyze the packages with tests that can simulate its treatment once at the market.

**Figure 9:**
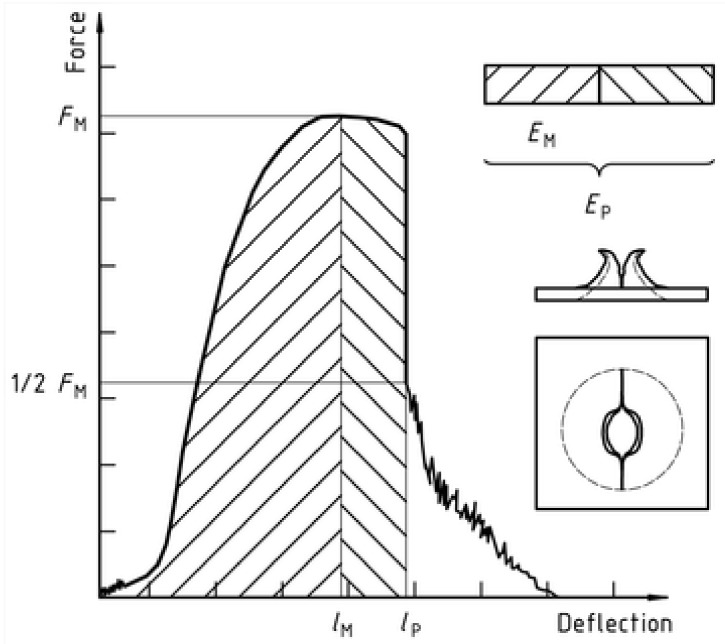
Example of force-deflection diagram for a ductile, with crease, specimen.

These tests are done on the industrially extruded sheet, before the thermoforming process. Registered curves for impact tests show a maximum which is related to the initial damage on the sheet, corresponding to the starting point of the fissure that will develop in a fracture. Analyzing the curves obtained in this test, maximum load, and its associated deformation can be known, as well as perforation energy. In Figure 9, it is shown an example of the force-deflection diagram obtained.

On the other hand, this test shows the way the sample breaks allowing us to define the failure mode of each specimen. Thickness is measure on extruded sheet, and the results shown in Table 6 are the average measures obtained in all the samples width (795 mm).

Results in Table 6 show that both the maximum load and perforation energy are lower in the nanocomposite samples than in the reference ones. Nanocomposites show fragile fracture and brittle behavior. This could be attributed, as well as explained for the laboratory results, to PET matrix degradation together with a poor PA_MXD6/nS particles dispersion, as seen in the MO pictures, and a high degree of crystallization of the PET matrix [9,13,25,26,27,28,29,30,31,32] due to the incorporation of the PA-nS particles. However, these results are better compared to those obtained in a laboratory extruder, probably due to a lower matrix degradation in the industrial extruder.

Comparing these results with those previously obtained by these authors [33] for a PET/nS sheet, in which the sepiolite was modified with AMEO as well, it can be observed there was a decrease in maximum force and puncture energy, indicating a PET matrix crystallization due to PA incorporation [24].

**Figure 10:**
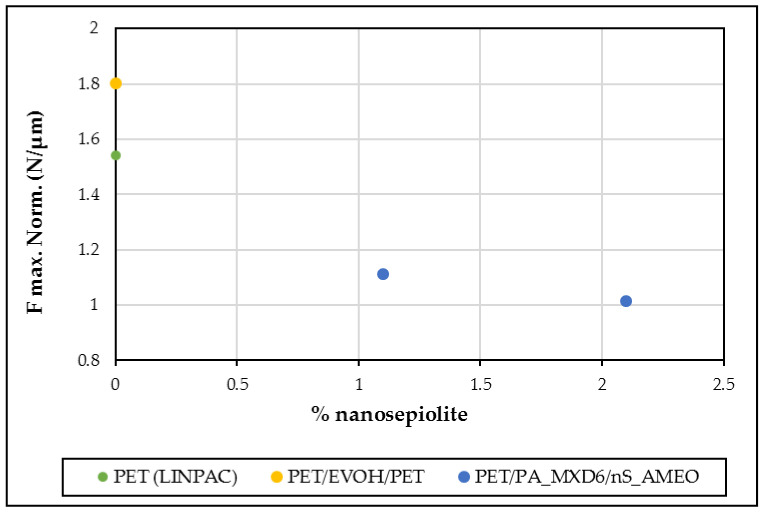
PET/PA_MXD6/nS_AMEO nanocomposites Normalized maximum force as a function of nanosepiolite content.

In order to compare results between themselves and compared them to the laboratory ones, the normalized force was calculated dividing by the sample thickness (Figure 10).

### 3.3. Microbiological Analyses and Shelf Life

**Figure 11:**
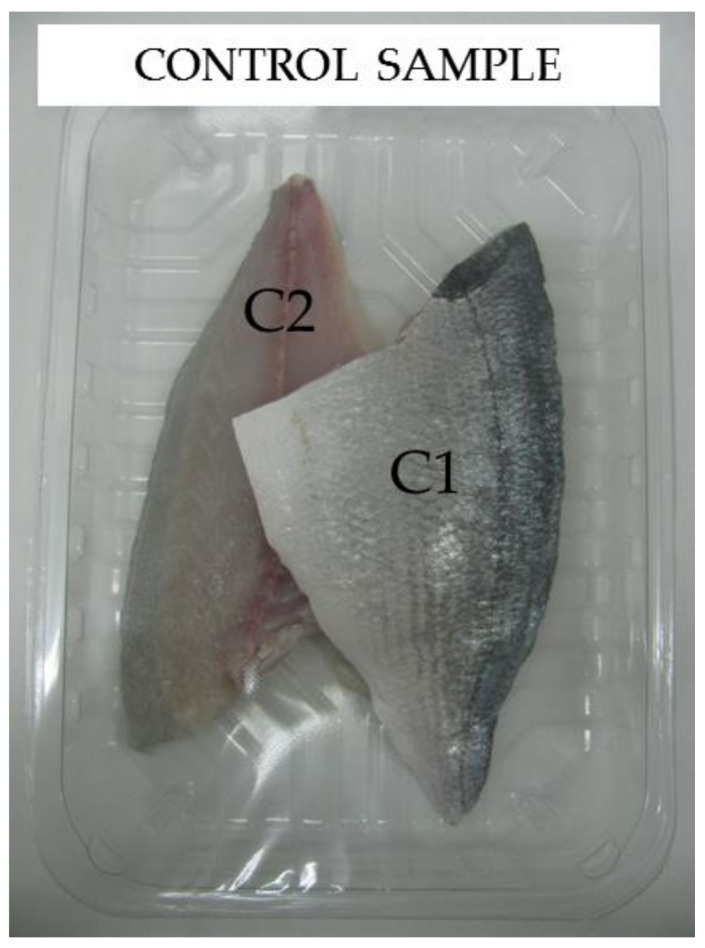
Control sample containing sliced sea bam.

As mentioned before, the tray used for these analyses are made with PET + 8% PA_MXD6 + 2.1% nS_AMEO and laminated with PE for sealing purposes. The reason for choosing this one was its better performance in permeability, together with its best processability on the industrial extruder. The control trays are made with PET/EVOH/PE material. The packed product is fresh and sliced sea bam, and the atmosphere inside the package is 40% N_2_ y 60% CO_2_ (Figure 11). CO_2_ is used for its bactericidal effect when used in MAP trays [4] and N_2_ act as a pressure regulator to compensate for the loss of CO_2_ due to fish meat absorption and through the package material. Microbiological parameters were measured on three samples of each sea bam fillet (with the same composition) during 9 days in order to count mesophilic aerobes and anaerobes.

The following tables (Table 7 and Table 8) show the average resulting mesophilic aerobes and anaerobes counts for each fillet and for the control samples, done on days 2, 4, 7, and 9.

Control of the samples should be done in accordance to Commission Regulation (EC) No. 2073/2005 [34]. It is not recommended, in fresh food, high number counts of mesophylls, although an elevated count does not imply the presence of pathogenic flora [19]. However, the total count reflects the sanitary quality of the analyzed products. At the same time, a low count does not mean the sample is pathogen free, it depends on the microbiota composition [35].

It is seen in the tables above that microbiological count is lower, by three orders of magnitude, in the tray produced with the new material (PET + 8% PA_MXD6 + 2.1% nS_AMEO). These results indicate an increase in shelf life of the fish in the nanocomposite tray compared to the PET/EVOH/PE one. For fresh water and marine species, the microbiological limit recommended by the ICMSF (1986) for APC at 30 °C is 7 log/g [23]. Based in these data, it is possible to say that shelf life of sea bream packed in this tray is longer than 10 days (approximately 13 days doing an extrapolation), whilst in PET/EVOH/PE trays its shelf life would be between 4 to 5 days.

**Figure 12:**
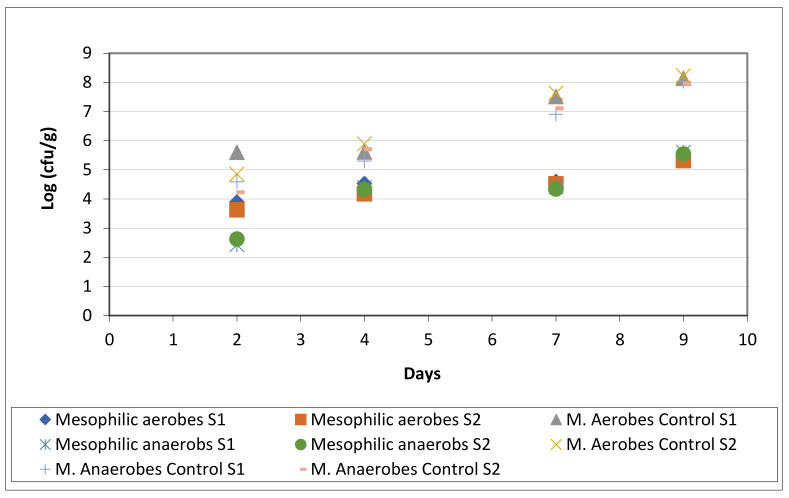
Microbiological count of sea bam packed in nanocomposite material and in PET.

In the following picture (Figure 12), it shows the evolution of the microbiological count during storage for both types of samples, PET/EVOH/PE control ones and those made with the nanocomposite. PET + 8% PA_MXD6 + 2.1% nS_AMEO. In this figure, it is clearly seen that the efficiency of the tested packaging material solution compared to the control one.

### 3.4. Migration

Migration analysis consist of testing plastic materials do not transfer their constituents to food. These analyses are regulated by the European Regulation EU No 10/2011 [36]. First of all, all the materials used during the plasic farication must be admitted in the positive list, that is to say, they need to be included in Regulation EC 975/2009 [37]. All the materials used in this work are registered in that list.

As per EU No 10/2011, plastic materials shall not transfer their constituents in quantitites exceeding the specific migration limits (SMI) or, in absence of these data, the generic migration limit of 60 mg/kg is applied. The SML is the maximum permitted quantity of a given substance that can migrate from a material or article into foodstuffs or food simulants. In these cases, testing is performed on the packaging simulating different situations, such as ambient temperature storage and freezing for several months or heating at diverse temperatures (these conditions are also regulated in EU No 10/2011). Moreover, these materials should not transfer their constituents to food simulants, in quantities over 10 mg of total constituents released per dm^2^ of food contact surface (global migration limit, GML).

GML tests have been done following specifications under UNE-EN 1186-14 [36]. For materials and articles not yet in contact with food verification of compliance with the overall migration limit is carried out in food simulants (defined in Annex III of the EU No 10/2011). The food simulant in this case is isooctane, used as a stable substitute of simulant D2 and the analyses are carried out during 10 days at 20 °C (OM1 conditions following regulations). Results in the nanocomposite trays, under those conditions, is <1.0 mg/dm^2^, thus it complies with global migration limits for the chosen simulant and under the test conditions.

Regarding SML, the substances in this work that have a SML are the silanes AMEO and MEMO. However, the final tray was produced using AMEO as organomodifier, thus results obtained for AMEO show a specific migration <0.04 mg/6 dm^2^, which is lower than its migration limit (0.05 mg/dm^2^). So, the tray complies with the limits stablished in Directive 2002/72/EC [37] and its subsequent amendments (Regulation EC 975/2009) for this product type. Actual legislation for food contact materials does not contain specific migration limits for natural silicates (except asbestos) such as sepiolite. However, since sepiolite is a magnesium silicate, it has been analyzed the global migration of silicon and magnesium in iso-octane, in order to know if there is any sepiolite migration from tray material to the packaged food. Results for silicon migration are lower than 0.05 mg/kg, and results for magnesium are lower than 1.0 mg/kg. For this reason, it is concluded that migration of these two elements to the packaged food is well under the legislation limit.

## 4. Conclusions

Results obtained with a nanocomposite sheet containing 2.1%nS (PET + 8% PA_MXD6 + 2.1% nS_AMEO) show an improvement in permeability of 43% and 34% against PET and PET/EVOH/PET sheets, respectively.

The main objective of this study was to investigate the influence of the nanocomposite packaging on the shelf life of the packed product. In this case, it has been achieved five days increase in the shelf life of packed sea bam when using PET/PA_MXD6/sepiolite nanocomposite as packaging material. The MAP trays used for keeping the fresh fish were produced industrially and meet the standards of migration for food contact products. Thus, these nanocomposite trays are useful to minimize the growth of contaminant microorganisms and further extend the shelf life of food while maintaining product quality and safety during storage.

## Figures and Tables

**Table 1:** Failure modes in the puncture impact test ISO 6603-2:2000 [22].

Specimen after Impact	Mode of Failure (Description)	Acronym
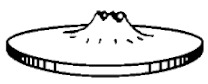	Ductile (creep with important reduction in the transversal section)	D
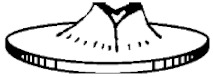	Ductile with crease (cracked towards the base)	Dc
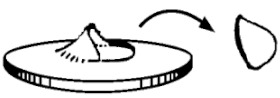	Transition Fragile/ductile (one or two pieces separated from the sample with a reasonable deformation)	FD
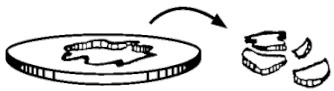	Fragile (more than one pieces separated from the sample, without any deformation)	F

**Table 2:** Percentage of nanosepiolite present in the samples.

Sample	rpm	% nS
PET/8PA_MXD6/1nS_AMEO	80	0.74
PET/8PA_MXD6/2nS_AMEO	80	1.98
PET/8PA_MXD6/1nS_AMEO	160	0.65
PET/8PA_MXD6/2nS_AMEO	160	1.74
PET/8PA_MXD6/1nS_MEMO	80	0.77
PET/8PA_MXD6/2nS_MEMO	80	1.57
PET/8PA_MXD6/1nS_MEMO	160	1.22
PET/8PA_MXD6/2nS_MEMO	160	1.78

**Table 3:** Percentage of nanosepiolite present in the masterbatches.

Sample	% nS
PA_MXD6/10nS_AMEO	12.4
PA_MXD6/20nS_AMEO	19.8

**Table 4:** Percentage of nanosepiolite present in the sheets.

Sample	% nS
PET/8PA_MXD6/1nS_AMEO	1.1
PET/8PA_MXD6/2nS_AMEO	2.1

**Table 5:** Permeability to O_2_ results of nanocomposite sheets.

Sample	% nS	Sheet Thickness (µm)	Permeability O_2_ (cm^3^/m^2^/day)	Permeability O_2_ (450 µm) ^1^ (cm^3^/m^2^/day)
PET (LINPAC)	-	450	8.2 ± 0.30	8.2 ± 0.30
PET/EVOH/PET	-	450	7.09 ± 0.11	7.09 ± 0.11
PET/8PA_MXD6	-	630	4.24 ± 0.15	5.93 ± 0.15
PET/PA_MXD6/nS_AMEO	1.1	440	5.38 ± 0.31	5.26 ± 0.31
PET/PA_MXD6/nS_AMEO	2.1	615	3.43 ± 0.10	4.68 ± 0.10

^1^ normalizing data to the same thickness.

**Table 6:** Impact puncture results of nanocomposite sheets.

Sample	% nS	Sheet Thickness (µm)	F Max (N)	E p	Failure Mode	Picture
PET (LINPAC)	-	467 ± 2	720 ± 16	6.6 ± 0.8	D	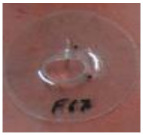
PET/EVOH/PET	-	555 ± 6	833 ± 12	7.0 ± 1.0	D	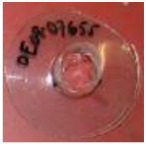
PET/PA_MXD6/nS_AMEO	1.1	440 ± 2	490 ± 11	1.1 ± 0.1	F	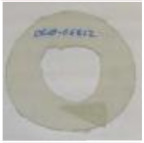
PET/PA_MXD6/nS_AMEO	2.1	611 ± 12	620 ± 70	1.2 ± 0.3	F	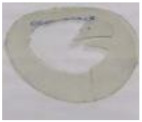

**Table 7:** Mesophilic aerobes count (ufc/g) in sliced sea bam packed in MAP.

Tray	S	Day 2	Day 4	Day 7	Day 9
PET + 8%PA_MXD6 + 2%nS_AMEO	S1	7.9 ± 0.25 × 10^3^	3.4± 0.17 × 10^4^	4.0 ± 0.12 × 10^4^	2.5 ± 0.20 × 10^5^
PET + 8%PA_MXD6 + 2%nS_AMEO	S2	4.3 ± 0.20 × 10^3^	1.5 ± 0.10 × 10^4^	3.3 ± 0.10 × 10^4^	2.1 ± 0.20 × 10^5^
Control	S1	4.0 ± 0.46 × 10^5^	4.1 ± 0.45 × 10^5^	3.3 ± 0.25 × 10^7^	1.4 ± 0.06 × 10^8^
Control	S2	7.2 ± 0.60 × 10^4^	7.9 ± 0.36 × 10^5^	4.3 ± 0.35 × 10^7^	1.7 ± 0.05 × 10^8^

**Table 8:** Mesophilic anaerobes count (ufc/g) in sliced sea bam packed in MAP.

Tray	S	Day 2	Day 4	Day 7	Day 9
PET + 8%PA_MXD6 + 2%nS_AMEO	S1	2.7 ± 0.17 × 10^2^	2.5 ± 0.17 × 10^4^	2.7 ± 0.17 × 10^4^	4.0 ± 0.17 × 10^5^
PET + 8%PA_MXD6 + 2%nS_AMEO	S2	4.2 ± 0.26 × 10^2^	2.1 ± 0.26 × 10^4^	2.2 ± 0.10 × 10^4^	3.5 ± 0.20 × 10^5^
Control	S1	3.9 ± 0.44× 10^4^	2.0 ± 0.43 × 10^5^	8.1 ± 0.72 × 10^6^	1.1 ± 0.19 × 10^8^
Control	S2	1.7 ± 0.36× 10^4^	5.1 ± 0.26 × 10^5^	1.3 ± 0.66 × 10^7^	9.0 ± 0.15 × 10^7^

## Data Availability

The data presented in this study are available on request from the corresponding author. The data are not publicly available due to privacy restrictions of the companies involved.

## Nomenclature

µm	Micrometer
AMEO	3-aminopropyltriethoxysilane
APC	Aerobic Plate Count
BOPET	Biaxially-oriented polyethylene terephthalate
cfu	Colony forming units
DSC	Differential Scanning Calorimetry
EFSA	European Food Safety Authority
Ep	Perforation Energy
EVA	Ethylene Vinyl Acetate
EVOH	Ethylene Vinyl Alcohol
F	Force
FCM	Food Contact Materials
FDA	Food and Drug Administration
h	Hours
IV	Intrinsic Viscosity
L/D	Relation length versus diameter
MAP	Modified Atmosphere Packaging
MAP	Modified Atmosphere Packaging
Max	Maximum
MEMO	3-methacryloxypropil trimethoxysilane
mm	Millimeter
MMT	Montmorillonite
nS	Nanosepiolite
OM	Optical Microscopy
OTR	Oxygen Transmission Rate
PA	Polyamide
PE	Polyethylene
PET	Poly(ethylene terephthalate)
rpm	Revolutions per minute
S	Sample
TGA	Thermogravimetric Analysis
